# Clinician Perspectives and Practices in Addressing Substance Use Among Children and Adolescents in NHS Grampian CAMHS

**DOI:** 10.1192/bjo.2025.10388

**Published:** 2025-06-20

**Authors:** Praveen Kumar, Caroline McKay, Nimisha Doval, Samuel Nixon

**Affiliations:** City Hospital, Aberdeen, United Kingdom

## Abstract

**Aims:** This study examines the perspectives, practices, and training needs of NHS Grampian CAMHS clinicians in addressing substance use among children and adolescents. The primary objectives are to:

Evaluate clinicians’ attitudes towards adolescent substance use.

Identify perceived barriers to effective assessment and intervention.

Inform the development of an integrated substance use pathway within CAMHS.

**Methods:** A cross-sectional survey was conducted between September and November 2023 using SNAP software. The survey was distributed to 48 CAMHS clinicians across NHS Grampian and included structured and free-text questions on demographics, clinical practices, perceived challenges, and training needs. Quantitative data were analysed in Microsoft Excel, while qualitative responses underwent thematic analysis.

**Results:** The survey captured diverse professional representation: 50% psychologists, 25% nurses, and 14.5% medical staff. While clinicians acknowledged substance use experimentation as part of normal adolescent development, they emphasized that persistent or problematic use requires structured intervention. The majority supported a multidisciplinary, multi-agency approach for better integration between mental health and substance use services.

Significant training gaps emerged: 46.9% of clinicians lacked familiarity with evidence-based interventions, while 40.8% required further training to implement them effectively. Only 10.2% reported regular use of such interventions in practice. Clinician confidence varied, with some expressing neutrality or disagreement regarding their preparedness to assess and manage problematic substance use.

Key barriers included limited resources, inadequate service integration, and challenges in engaging young people and families. Qualitative responses highlighted the need for structured training, clearer referral pathways, and enhanced service coordination. Clinicians emphasised the importance of ongoing education programmes that evolve with emerging substance use trends.

**Conclusion:** The findings reveal significant gaps in clinician training, confidence, and service integration. Recommendations include:

Expanding training opportunities to strengthen familiarity with evidence-based interventions.

Enhancing referral pathways to improve integration with substance use services.

Developing engagement strategies to support young people and families in accessing treatment.

These findings highlight the need to improve clinicians’ awareness, confidence and training working with young people who use substances.

